# The role of cryptocurrencies in predicting oil prices pre and during COVID-19 pandemic using machine learning

**DOI:** 10.1007/s10479-022-05024-4

**Published:** 2022-10-28

**Authors:** Bassam A. Ibrahim, Ahmed A. Elamer, Hussein A. Abdou

**Affiliations:** 1 Department of Management, Faculty of Commerce, Mansoura University, Mansoura, Egypt; 2 Brunel Business School, Brunel University London, Kingston Lane, Uxbridge, London, UB8 3PH UK; 3 Departmentof Accounting, Faculty of Commerce, Mansoura University, Mansoura, Egypt; 4 Faculty of Business & Justice, University of Central Lancashire, Preston, PR1 2HE UK

**Keywords:** Cryptocurrencies, COVID-19, Bitcoin, Machine learning, Crude oil, Neural networks

## Abstract

This study aims to explore the role of cryptocurrencies and the US dollar in predicting oil prices pre and during COVID-19 pandemic. The study uses three machine learning models (i.e., Support vector machines, Multilayer Perceptron Neural Networks and Generalized regression neural networks (GRNN)) over the period from January 1, 2018, to July 5, 2021. Our results are threefold. First, our results indicate Bitcoin is the most influential in predicting oil prices during the bear and bull oil market before COVID-19 and during the downtrend during COVID-19. Second, COVID-19 variables became the most influential during the uptrend, especially the number of death cases. Third, our results also suggest that the most accurate model to predict the price of oil under the conditions of uncertainty that prevailed in the world during the bear and bull prices in the wake of COVID-19 is GRNN. Though the best prediction model under normal conditions before COVID-19 during an uptrend is SVM and during a downtrend is GRNN. Our results provide crucial evidence for investors, academics and policymakers, especially during global uncertainties.

## Introduction

The global COVID-19 pandemic has created economic chaos worldwide and made a number of severe socio-economic issues (Abedin et al., 2021; Ftiti et al., 2021; Queiroz et al., 2020). For instance, governmental restrictions such as large-scale closure and travel restrictions due to lockdown actions led to an unprecedented decline in global growth by 3.2% in 2020 (Abedin et al., 2021). Due to these severe uncertainties, many investors moved to secure safe-haven assets because trade risky assets have the largest decline in a single week since the 2008 global financial crisis in the final week of February 2020 (Park, 2022). Also, the Dow Jones Industrial Average (DJIA) declined by 33% on 20 March 2020, from its 2019 value, since the start of COVID-19 in Wuhan, China (Abedin et al., 2021; Albitar et al., 2021; Alshater et al., 2022; Chen et al., 2021; Elmarzouky et al., 2021). This has led to a quick decline in the global demand for crude oil with sharp downward variations. For example, West Texas intermediate (WTI) oil prices have crushed to below zero in April 2020, with a 4.5% drop in the world industrial production index in the first three months of 2020 (Salisu et al., 2021). A strand of literature has been conducted on crude oil because it is one of the most vital commodities worldwide. It represents about 50% of the general commodity index (Bašta & Molnár, 2018). Also, it has become evident that crude oil works as a fundamental asset in the trading of different financial instruments and the expanded power of oil price shocks on the global financial markets.

Meanwhile, cryptocurrency markets have been affected and gained investors' attention during the current COVID-19 uncertainties. Bitcoin and Ethereum, which are the greatest illustrative cryptocurrencies, have documented the biggest trading volumes and occupy the highest market capitalizations (Kim et al., 2021). For instance, Bitcoin has noted a trading volume of USD 1240 billion (21,336,435 BTC) and a market capitalization of USD 1097 billion, and Ethereum has reached a trading volume of USD 546 billion (118,187,782 ETH) and a market capitalization of USD 547 billion in November 2021. It is worth mentioning that Bitcoin and Ethereum account for 62% of the cryptocurrency markets according to the CoinMarketCap data,^1^ Consequently, Bitcoin and Ethereum were chosen to represent cryptocurrencies. The global COVID-19 outbreak has affected cryptocurrency markets. For instance, the biggest weekly decline in the Bitcoin price (nearly 36%) occur on 13 March 2020 (Jareño et al., 2021). Although these declines paralleled oil prices, we have observed the opposite in other periods. Specifically, Fig. 2 shows that oil prices were rising and Bitcoin and Ethereum prices were falling and vice versa during periods from October 2019 until August 2021. Academic literature argues that cryptocurrencies such as Bitcoin can be used for hedging against oil, especially during times of political and economic turmoil (Al-Yahyaee et al., 2019; Das et al., 2020; Ghazani & Khosravi, 2020; Mo et al., 2018; Selmi et al., 2018). Thus, cryptocurrency assets are commodities and present the same aspects of commodity markets. Per se, we expect that cryptocurrency prices may predict global crude oil prices. Moreover, active trading and mining of cryptocurrencies demand extensive electricity consumption. This may affect the energy markets (Okorie & Lin, 2020). Thus, our study aims to predict crude oil prices using cryptocurrencies and the US dollar pre and during COVID-19 in times of severe uncertainty. Then, it determines the importance of these variables in predicting the price of oil before and after COVID-19 and identifying the most accurate neural network model during the market downtrend and uptrend.

Using three neural network models (i.e., SVM, MLP and GRNN), we predicted the price of USOIL based on historical data for Bitcoin, Ethereum, the US dollar index and the COVID-19, dividing our data into 8 scenarios before and during COVID-19 with and without COVID-19 variables during the uptrend and downtrend of oil markets. Our results indicate that Bitcoin is the most influential in predicting oil prices during the bear and bull oil market before COVID-19 and the downtrend during COVID-19. Ethereum has become the most influential during the bull oil market during COVID-19. The reason for this may be due to Tesla cancelling dealing in Bitcoin and the statement of its chairman that the reason for this is the use of fossil fuels in mining. In addition, Bitcoin has been banned in China during this period. After adding COVID-19 variables to our model, we found that they became more important than Ethereum and the US dollar index during the downtrend, and Bitcoin continued to be the most influential according to SVM and MLP, while COVID-19 variables became the most influential during the uptrend, and the most influential variable was death cases according to the three models. Our results also suggest that the most accurate model to predict the price of oil under the conditions of uncertainty that prevailed in the world during the downtrend during COVID-19 is GRNN and during the uptrend also if the COVID-19 data is used as a total case alone, but if we add the new cases, the most accurate model is SVM. Though the best prediction model under normal conditions before COVID-19 during an uptrend is SVM and during a downtrend is GRNN.

This study contributes to the current literature in several ways. First, we use machine learning (Karim et al., 2021; Kazancoglu et al., 2022; Khalilpourazari & Hashemi Doulabi, 2022) to understand the predictability power of cryptocurrencies, the US dollar, and the COVID-19 on oil prices. Other studies used conventional models to investigate this relationship (e.g., Albulescu & Ajmi, 2021; Bénassy-Quéré et al., 2007; Charfeddine et al., 2020; Jareño et al., 2021; Mensi et al., 2020; Okorie & Lin, 2020; Kumar et al., 2022a, 2022b; Kumar et al., 2022a, 2022b; Nyawa et al., 2022; Queiroz et al., 2020; Queiroz & Fosso Wamba, 2021; Wen et al., 2018; Zhang et al., 2008). Second, we cover a longer period during COVID-19. Specifically, we cover the period from January 2020 to July 2021, which is a longer period than the period covered by previous literature that studied cryptocurrencies and oil during COVID-19 (Jareño et al., 2021). Third, to the best of our knowledge, this is the first work that describes the importance of the impact of each variable of Bitcoin, Ethereum and the US dollar in predicting oil prices in detail before and during COVID-19. Fourth, to the best of our knowledge, this is the first work that includes specific and detailed COVID-19 variables such as total confirmed, total death, total recovered, new confirmed, new death and new recovered cases. Fifth, to the best of our knowledge, this is the first work that describes the effect of cryptocurrencies, the US dollar and COVID-19 on oil during different uncertain periods such as the up- and down-market trends. Sixth, we identify the most accurate model that can be used before and during COVID-19 in the up-and-down-market trends. In other words, we identify the most accurate prediction model under normal and severe uncertainties conditions.

The remainder of this study is constructed as follows: Sect. 2 reviews the literature. Section 3 describes our dataset and methodology. Section 4 presents and discusses our results while Sect. 5 concludes our work.

## Literature review and hypotheses development

This section analyses extant literature on predicting oil prices and offers the theoretical reasoning for examining the impact of cryptocurrencies and COVID-19 on the predictability of oil prices.

### Cryptocurrency and oil prices

**Fig. 1:**
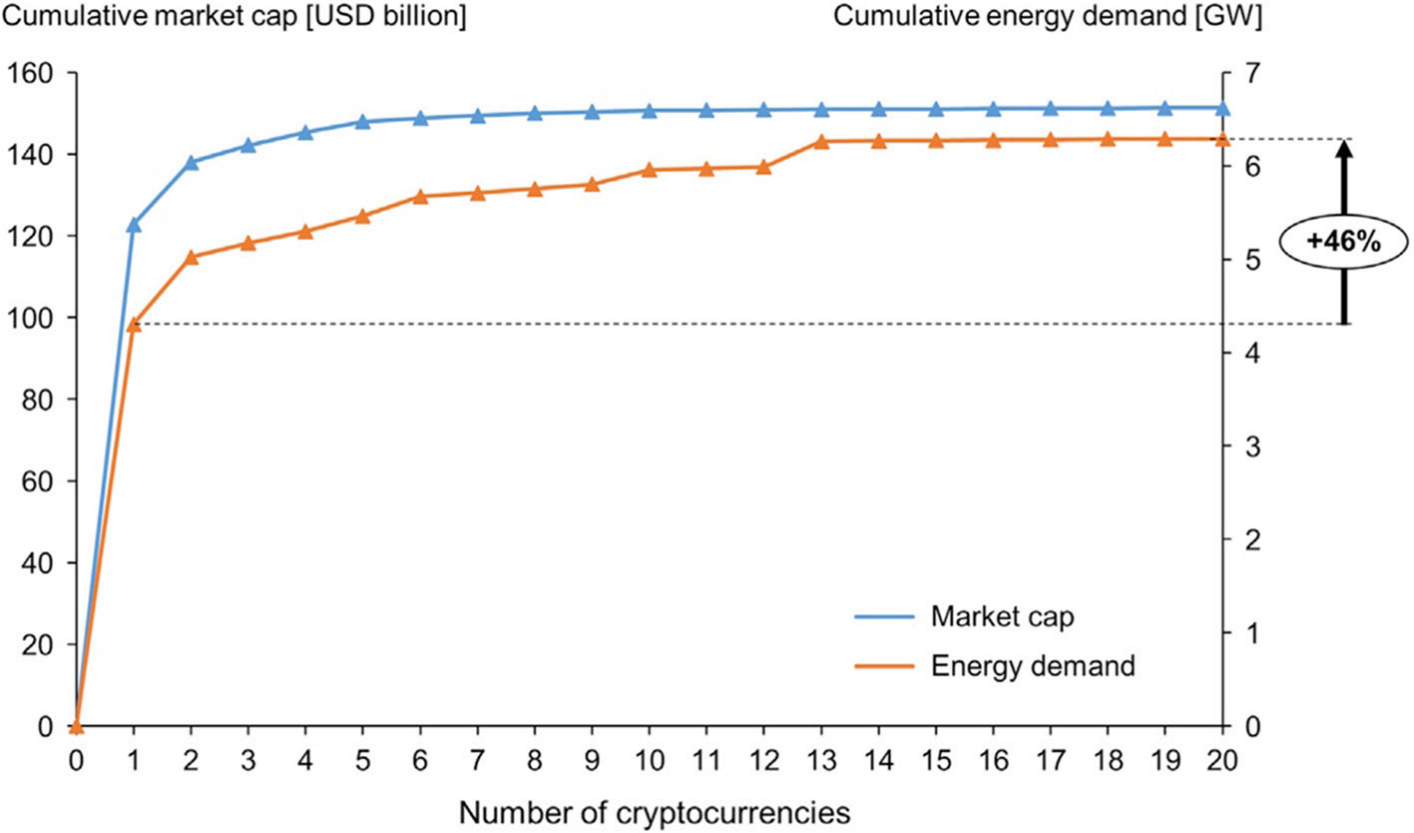
Cumulative Market Capitalization and Energy Demand of Top 20 Currencies by Market Capitalization. Source: Gallersdörfer et al. (2020), p 1845

A growing strand of literature recently has focused on the analysis of cryptocurrencies with the aim of discovering the response of the cryptocurrency markets to the COVID-19 outbreak in addition to differences in the relations between cryptocurrencies and other traditional assets (Corbet et al., 2019; Jareño et al., 2021). Corbet et al. (2019) review the existing literature, suggesting that cryptocurrencies are reliable investment assets with genuine value. several studies examined the potential relation between energy and cryptocurrencies from the view of the influence of energy prices on cryptocurrency prices (Bouri et al., 2017a, 2017b; O’Dwyert & Malone, 2014). These studies show that energy is associated with Bitcoin and other cryptocurrencies. In fact, cryptocurrencies production depends on mining, which consumes a lot of energy. This is clear in the case of Bitcoin (Bouri et al., 2017a, 2017b). O’Dwyert and Malone (2014) expect that electricity utilized in Bitcoin mining is almost equal to Ireland electricity consumption. Li et al. (2019) also show that Monero mining electricity consumption in the world in 2018 is 645.62 GWh worldwide. Gallersdörfer et al. (2020) show that the biggest cryptocurrency from the market capitalisation view (i.e., Bitcoin) is responsible for 2/3 of the total energy demand, while the second cryptocurrency from the market capitalisation view (i.e., Ethereum) accounts for 11.46% of the total energy demand as shown in Fig. 1.

Hayes (2017) indicates that Bitcoin value shows the mining cost. Kristjanpoller and Minutolo (2021) examined and provided evidence about the fractal and cross-correlation between electricity prices in the USA and crude oil and natural gas prices. Therefore, an increase in oil prices signals a potential increase in Bitcoin prices. Yet, this relationship might be bidirectional. On the one hand, cryptocurrencies such as Bitcoins are generated by utilizing intensive energy and, as a result, lower energy prices may lead to lower Bitcoin prices. This suggests a positive association between energy and Bitcoin prices (Bouri et al., 2017a, 2017b).

On the other hand, other studies have examined cryptocurrencies such as Bitcoins as hedging tools. Al-Yahyaee et al. (2019), Bouri et al., (2017a, 2017b) and Dyhrberg (2016) suggest that investors can use cryptocurrencies such as Bitcoins as a hedge against uncertainty. Also, Bitcoins can be employed as a hedge against the US dollar in the short term. Thus, Bitcoin has some hedging abilities like traditional hedging commodities such as gold and can be used to hedge market-specific risk. in the same vein, Das et al. (2020) explore the hedging and safe haven characteristics of Bitcoin against crude oil implied volatility and structural shocks. They found that traditional hedging commodities such as gold, commodity and the US Dollar outperform Bitcoin to hedge oil-related uncertainties. However, Selmi et al. (2018) suggested that despite that Bitcoin can be employed as a hedging instrument, it depends on Bitcoin's different (bear, normal or bull) market conditions and the trend of oil prices. Also, Guesmi et al. (2019) show that Bitcoin can be employed for hedging. They mentioned that Bitcoin will be the best option to lower the overall portfolio risk if investors need to add other assets to their portfolio of gold, oil and equities.

In the same context, other studies examined Bitcoin as a diversifier. Charfeddine et al. (2020) show that cryptocurrencies can act as financial diversification. Moreover, Charfeddine et al. (2020) find that the association between cryptocurrencies and conventional assets (gold, S&P 500, and oil) is vulnerable to outside economic and financial shock waves. Dutta et al. (2020) show that Bitcoin acts only as a diversifier for crude oil but not as a safe haven. Al-Yahyaee et al. (2019) support this notion. In the same line, a strand of the literature suggests that Bitcoin is less effective than the traditional safe-haven asset such as gold when it acts as a safe-haven (Baur et al., 2018; Klein et al., 2018; Musialkowska et al., 2020). Smales (2019) points out that liquidity problems, transaction costs and the time required to execute transactions hinder Bitcoin from being an optimal safe-haven asset.

Regarding the relationship between cryptocurrencies and oil, Gajardo et al. (2018) suggest that Bitcoin is greater multifractal spectra compare to the other currencies with crude oils (WTI). Ghazani and Khosravi (2020) support this notion and found the cross-correlations between three cryptocurrencies (including Bitcoin, Ethereum and Ripple) and crude oils (WTI and Brent). Van Wijk (2013) shows a negative relationship between Bitcoin and oil prices. Huynh et al. (2020) show that the USA and European crude oil indices shocks are largely associated with the movements of most cryptocurrencies. Further, the findings show that the European crude oil prices are a source of shocks to the cryptocurrencies while the USA oil index looks to be a receiver of shocks. On the contrary, other studies have pointed to the impact of cryptocurrencies on oil, for example (Ji et al., 2019) study the information interdependence between major cryptocurrencies and some commodities such as energy, agriculture, and metals. It finds that the interdependence changes over time as cryptocurrencies are becoming more connected and prominent over time while energy commodities are dependent on cryptocurrencies' price dynamics.

**Fig. 2:**
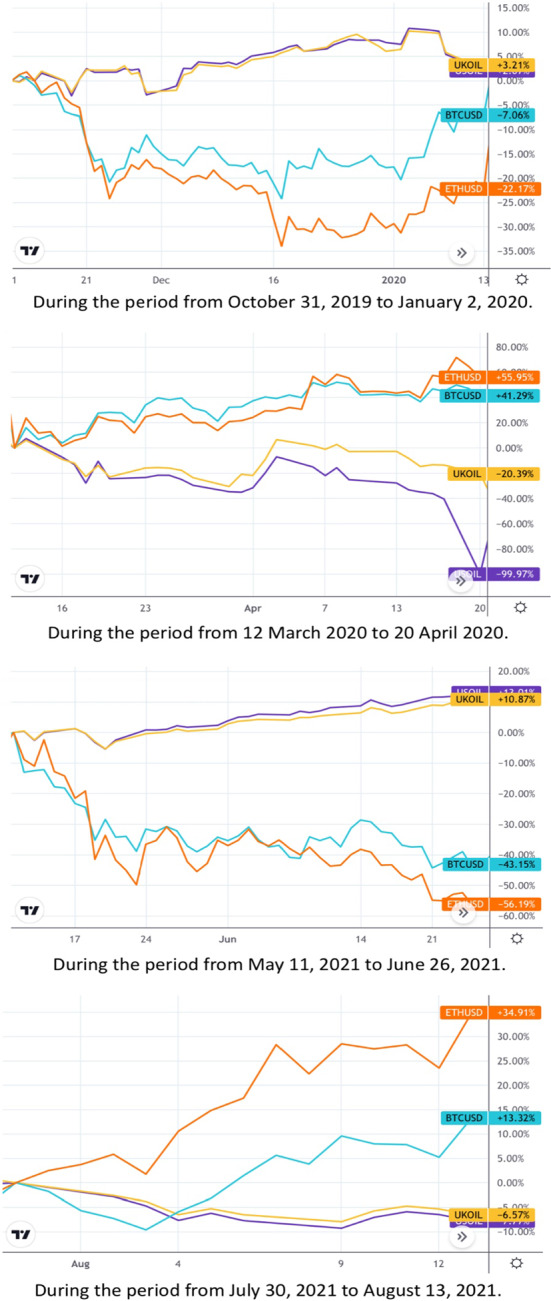
The inverse relationship between USOIL and UKOIL on one side and Bitcoin and Ethereum on the other side. Variables are defined in Appendix 1 Source: Tradingview

According to the above previous studies, cryptocurrencies can be used as diversifiers (Al-Yahyaee et al., 2019; Charfeddine et al., 2020; Dutta et al., 2020) or hedging (Al-Yahyaee et al., 2019; Bouri et al., 2017a, 2017b; Das et al., 2020; Dyhrberg, 2016; Guesmi et al., 2019; Selmi et al., 2018) or it can also be a safe haven (Klein et al., 2018; Musialkowska et al., 2020). Consequently, when there is an expectation of the arrival of a period of uncertainty, mutual funds, hedge funds and individual investors will turn to a safe haven, hedge or diversify to face this period, which will lead to a rise in cryptocurrencies price and then a decline in oil prices as they approach a period of uncertainty. That an increase in cryptocurrency prices occurs before the fall in oil prices, as is the case in the government bond market, and we can infer what happened during the Covid-19 pandemic, which is shown in Fig. 2. It suggests that the price of both Bitcoin and Ethereum increases while the price of oil decreases.

Most of the previous studies focused on the impact of oil prices on cryptocurrency prices, as cryptocurrencies consume energy to mine them (Bouri et al., 2017a, 2017b; Hayes, 2017; Krugman & Obstfeld, 2003; Palombizio & Morris, 2012), but few have studied otherwise (Ji et al., 2019) and we add to this rare literature, as we study the impact of cryptocurrencies on oil prices. What motivated us for that is three reasons: First: cryptocurrencies are used by investors, investment funds and hedging funds for diversification, hedging and as a safe haven. Therefore, the demand for it can increase during periods of uncertainty, and consequently, the profits of the miners, which may push more miners to enter the cryptocurrencies mining industry and the current miners increase their activity, which may lead to an increase in demand for energy and thus increase the demand for oil that is used in power generation and thus increases its prices. Second: investment fund managers and investors may expect a state of uncertainty in the future, which may push them to buy cryptocurrencies to face this situation, and thus the change in cryptocurrencies prices may precede the change in oil prices. Third, a scarcity of previous studies that studied the impact of cryptocurrencies on oil. Yet, to our best knowledge, no extant literature has focused on the relation between the cryptocurrencies market and oil prices during the COVID-19 pandemic using machine learning. Thus, we hypothesize that:

#### H1

Machine learning models can enhance the predictability of cryptocurrencies on oil prices pre and during the COVID-19 outbreak.

### USD and oil prices

A number of theories explain the relationship between oil prices and exchange rates (Albulescu & Ajmi, 2021). Darby (1982) refers to the supply–demand avenue as oil price differences are associated with greater inflation. Specifically, national interest rate changes due to inflationary pressures, thus influencing the national currency value in line with the real interest rate parity hypothesis. Bénassy-Quéré et al. (2007) assert that short-run portfolios and the medium- and long-run wealth channels influence a currency increase for the oil-exporting nations, creating a currency devaluation of oil-importing countries. The exchange rate influence on oil prices is likewise happening as a result of oil prices are generally designated in USD. The decrease of one rate indicates that the USD increase lessens the demand for oil beyond the USA (Blomberg & Harris, 1995). Additionally, the USD increase may create growth in oil supply, causing a reduction in oil prices at the global level (Coudert et al., 2007).

We found many results from empirical studies that indicate the existence of a relationship between the exchange rate of the USA dollar and oil as follows. Ferraro et al. (2015) show evidence to establish the presence of a short-term connection between the nominal Canadian-USA exchange rate and simultaneous oil prices. The findings indicate that oil prices can forecast the exchange rate at a daily rate. While Ding and Vo (2012), Fratzscher et al. (2014) and Wu et al. (2012) observed a bidirectional connection between the USD and oil prices. Likewise, Aloui et al. (2013) noticed that the decrease in USD is related to crude oil price growth, especially during the global financial crisis. Cifarelli and Paladino (2010) and Jiang and Gu (2016) also document a negative association between the volatility of real oil prices and the real USD exchange rate in the long run.

Other studies found an effect of oil on exchange rates as follows: Wen et al. (2018) suggest a nonlinear Granger-cause of the USD exchange rate and crude oil prices, but not vice versa. Turhan et al. (2013) use daily data to investigate the role of oil prices in describing the underlying forces of the exchange rate in developing countries. Turhan et al. (2013) show that an increase in oil prices leads to a considerable increase in currencies versus the USD. Likewise, Lizardo and Mollick (2010) utilized a cointegration test and discovered that oil prices impact substantially in justifying long-term USD movements.

Alternatively, further studies have discovered an influence of the USD exchange rate on oil prices. Mo et al. (2018) examine and show the dynamic negative linkages between the US dollar and the crude oil market, however, after the global financial crisis they see a positive non-linear correlation from USD to crude oil. In the same line, Houcine et al. (2020) examine and found a co-integration connection between the price of crude oil in USD per barrel, and the Euro-Dollar exchange rate using Auto-Regressive Distributed Lag (ARDL). Granger causality test result reveals a one-way correlation between the Euro-Dollar exchange rate towards oil prices; specifically, the change in the exchange rate leads to fluctuations in oil prices. Sadorsky (2000) suggests that futures prices for crude oil and oil are related to exchange rates. Akram (2009) indicates that a lower USD causes greater commodity prices. Shocks also to the dollar are observed to represent significant shares of variations in commodity prices. Zhang et al. (2008) show that there is a substantial long-term equilibrium cointegration connection between the US dollar exchange rate and international crude oil prices. Wen et al. (2018) suggest that the USD exchange rate offers a greater and more stable negative impact on crude oil prices in the short term, and the effect steadily declines after 2012. Similarly, Yousefi and Wirjanto (2004) and Lin et al. (2016) show similar results.

Based on the above argument and results, we find that there is a potential relationship between the USD and oil prices (Aloui et al., 2013; Cifarelli & Paladino, 2010; Ding & Vo, 2012; Ferraro et al., 2015; Fratzscher et al., 2014; Jiang & Gu, 2016; Wu et al., 2012), and there is evidence that oil affects the USD (Lizardo & Mollick, 2010; Turhan et al., 2013; Wen et al., 2018) and vice versa (Akram, 2009; Houcine et al., 2020; Lin et al., 2016; Mo et al., 2018; Wen et al., 2018; Yousefi & Wirjanto, 2004). Thus, we use three models named: Support Vector Machine (SVM), General Regression Neural Networks (GRNN) and Multilayer Perceptron Networks (MLP) to study the impact of cryptocurrencies and the US dollar on oil prices before and during the COVID-19 pandemic, during the up and down market trends, and their ability to predict oil prices and determine the importance of each of them in influencing oil. Thus, we hypothesize that:

#### H2

Machine learning models can enhance the predictability of the US dollar on oil prices pre and during the COVID-19 outbreak.

## Research design

### Experimental data

**Fig. 3:**
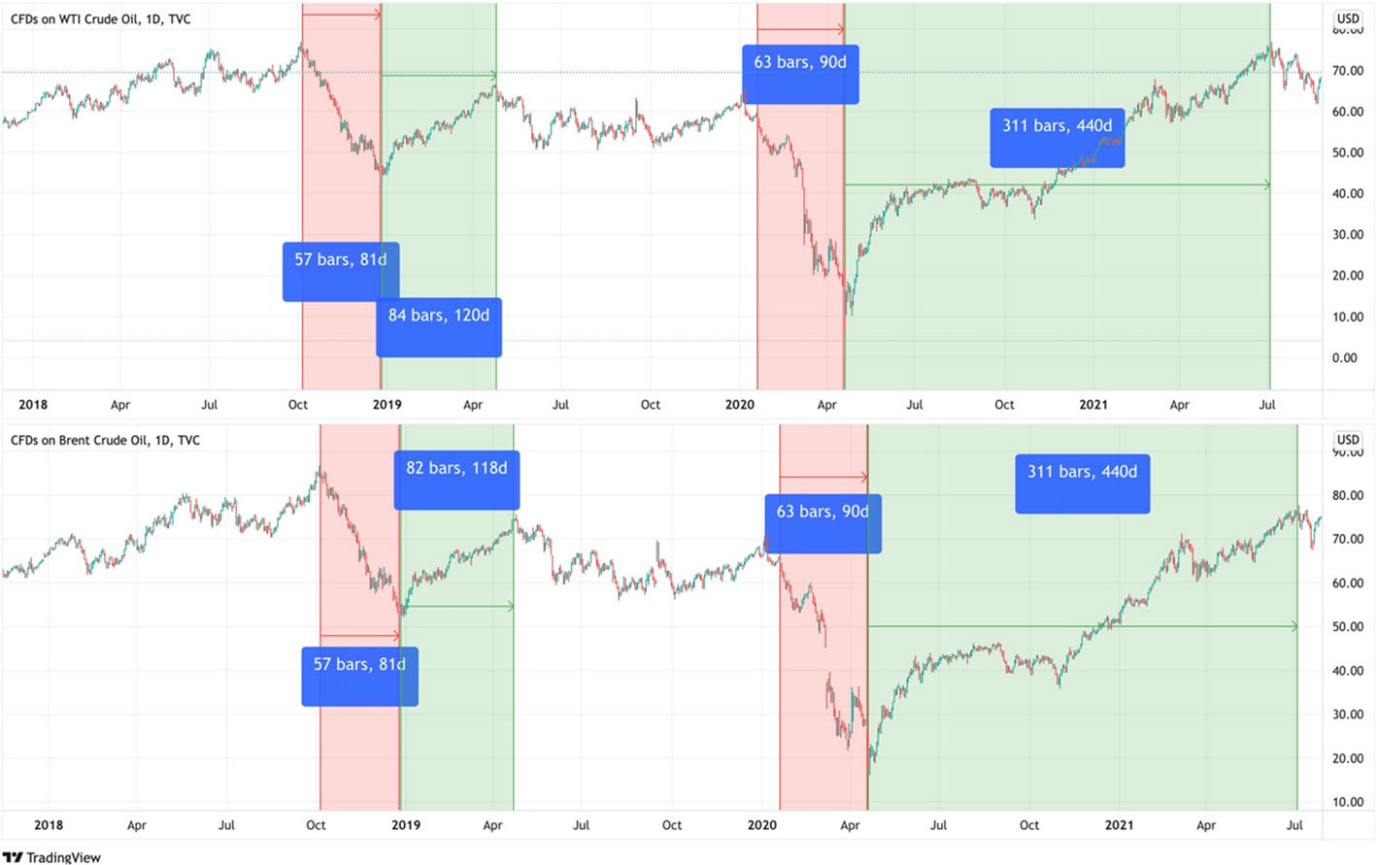
The uptrend and downtrend of USOIL and UKOIL before and during COVID-19. Variables are defined in Appendix 1. Source: Tradingview

The dataset came from TradingView.^2^ The data is the daily closing of West Texas Intermediate CFDs (USOIL). CFDs were used as they are traded 24 h a day and 5 days a week, including during the close of the main markets to reflect fresh COVID-19 announced data, which is used in the baseline analysis. While, daily closing of Brent crude oil CFDs was used in a robustness check and the daily closing of the US dollar index (DXY) and daily COVID-19 data worldwide including confirmed, death, and recovered cases. The data covers the period from January 1, 2018, to July 5, 2021. Data panels were divided based on USOIL and UKOIL price trends and the existence of the COVID-19 pandemic. According to technical analysis, prices move in an uptrend, downtrend or horizontal trend (Murphy, 1999). Figure 3 shows that there is a downtrend (in red area) in USOIL and Brent crude oil (UKOIL) prices at the beginning of COVID-19. Then prices changed to an uptrend (in green area) and there was no horizontal trend during this period. Consequently, the last uptrend and downtrend before COVID-19 were chosen to compare the model's results during the downtrend before and during COVID-19 and its results during the uptrend before and during COVID-19.

We employ the most representative cryptocurrencies that are Bitcoin and Ethereum. Specifically, both cryptocurrencies have recorded the largest trading volumes and possess the highest market capitalizations (Kim et al., 2021). As of November 2021, Bitcoin has recorded a trading volume of USD 1240 billion (21,336,435 BTC) and a market capitalization of USD 1097 billion, and Ethereum has reached a trading volume of USD 546 billion (118,187,782 ETH) and a market capitalization of USD 547 billion. It is worth noting that Bitcoin and Ethereum control 62% of the cryptocurrency market, according to the data on the CoinMarketCap website.^3^ Thus, Bitcoin and Ethereum were chosen to represent cryptocurrencies.

### Methodology

In building our models, DTREG software is used. Three different machine learning modelling techniques are used, namely, Support Vector Machines (SVM); Multilayer Perceptron Neural Networks (MLP) and Generalized Regression Neural Networks (GRNN).

#### Support vector machines

Support vector machines (SVM) are a common category of supervised machine learning algorithms. They are a comparatively new modelling technique that showed potential at building accurate models for a variety of problems and are closely related to neural networks. SVM is predominantly good at pattern recognition, but it is also applicable to various types of modelling applications (DTREG, 2021). An SVM model which uses ‘sigmoid kernel function’ can be equivalent to a two-layers multilayer perceptron (also known as feed-forward) neural network or ‘radial basis function’. It can be used for both classification and regression modelling problems. Furthermore, it performs the model by constructing an N-dimensional ‘hyperplane’ that optimally splits data into two (i.e., binary dependent variable) or more (continuous dependent variable). SVM uses quadratic programming problems with linear constraints to solve the wight of the network.

In an SVM network ‘a predictor variable is called an attribute, and a transformed attribute that is used to define the hyperplane is called a feature’ (DTREG, 2021, p. 289). Then ‘feature selection’ is taken place where the most appropriate representation of data is chosen. Each set of ‘features’ describes one raw of predictor values and is called a ‘vector’. Therefore, SVM modelling works in a way to find the optimal ‘hyperplane’ that separates clusters of ‘vectors’. Those vectors which are near the hyperplane are called ‘support vectors’. In this paper, we use the ‘radial basis function’ as the recommended kernel function in building our SVM models (DTREG, 2021).

#### Multi-Layer perceptron neural network

**Fig. 4:**
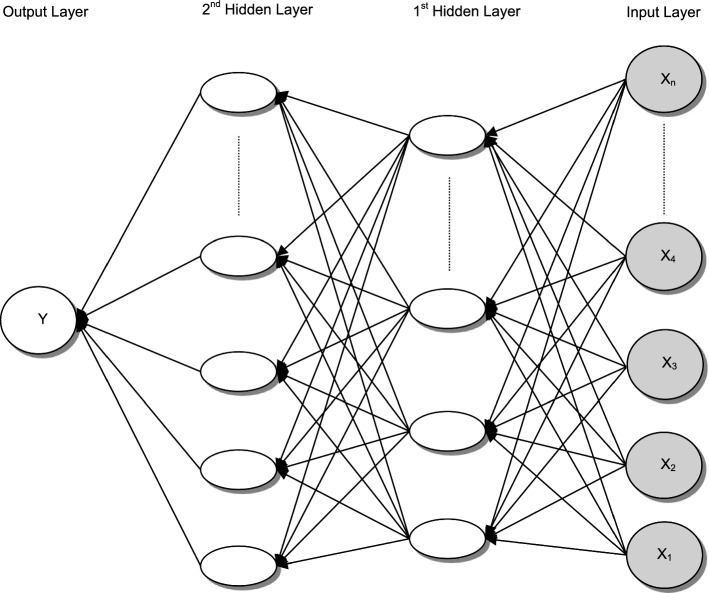
Architecture of a Multi-layer Perceptron Neural Network *Note:* This figure presents a structure for MLP. In this network, the number of nodes in the 2nd hidden layer is larger than the number of nodes in the 1st hidden layer. The output at a given layer (e.g., the 2nd hidden layer) can be expressed as a connection-weighted summation of outputs from the previous layer (e.g., 1st hidden layer) plus a neuron bias (a parameter assigned to each neuron). Arriving at a neuron in the output layer, the value from each hidden layer neuron is multiplied by a weight, and the resulting weighted values are added together. Finally, $Y$ values are produced by a conversion function for the output layer (Abdou et al., 2019, p. 5; Abdou, 2009, p.101; modified)

Multilayer Perceptron Neural Networks (MLP) also known as Multilayer Feed-forward Neural Networks were initially developed by Frank Rosenblatt (1958) and are more suitable to be used to describe complex relationships between independent predictor variables (Abdou, et al., 2019). Figure 4 presents an example of an MLP architecture.

#### Generalised regression neural networks

**Fig. 5:**
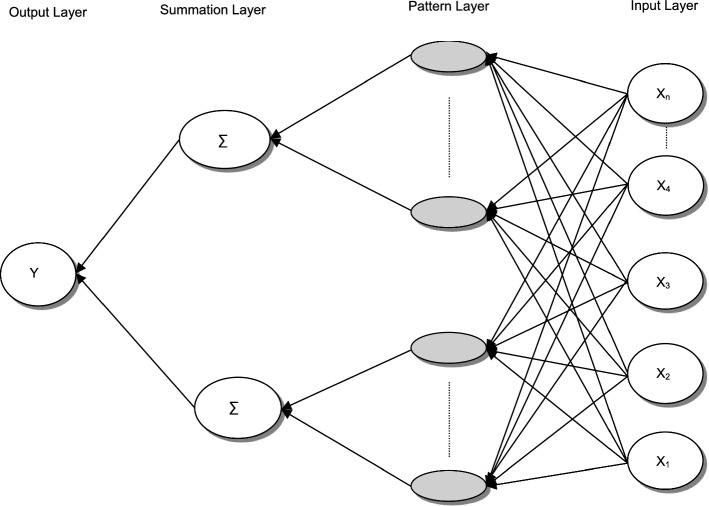
Architecture of Generalized Regression Neural Network *Note*: This architecture presents four GRNN layers. The 1st layer i.e., input layer comprises a neuron for each independent predictor variable in the model. Each node in the 2nd layer i.e., pattern layer, which contains one node for each training case, measures the distance between each of the input values and the training values reintroduced by each of the nodes. Then, each of these values pass to each of the nodes in the 3rd layer i.e., summation layer (Numerator & denominator nodes), which is a function of the distance in the smoothing factors. One node per dependant predictor variable is in the 3rd layer, each node computes a weighted average using the training cases in that category. In the 3rd layer i.e., summation layer, the nodes sum its inputs, whilst the output node divide then to generate the best possible predictions (Abdou, et al., 2021, p. 6285; Abdou, et al., 2012, p. 800)

Generalised Regression Neural Network (GRNN) is a network with similar architecture to a Probabilistic Neural network but with the essential difference that GRNN runs regression with a continuous dependent variable. Both Networks are theoretically like k-Nearest Neighbour known as k-NN but with completely different applications. Furthermore, Abdou et al., (2012, 2021) explained that GRNN does not require various stationarity tests that regression family models would require. Figure 5 presents an example of a GRNN architecture.

## Empirical results and discussion

### The predictability power of cryptocurrencies, the US dollar and COVID-19 on oil prices.

Oil price prediction has been made for four different scenarios. Two of those scenarios are the downtrend and uptrend periods before the COVID-19 outbreak. The remaining scenarios are the downtrend and uptrend during the COVID-19 outbreak.

#### Scenario 1: Results during downtrend before COVID-19 outbreak

**Fig. 6:**
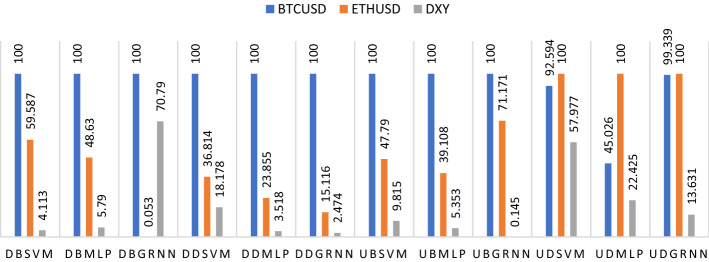
Ranking of cryptocurrencies and the US dollar according to their importance in affecting oil before and during COVID-19. Variables are defined in Appendix 1

When we run the model during the downtrend before covid-19, there is an agreement between SVM, MLP and GRNN models on the results of measuring the predictability power of cryptocurrencies and the US dollar (DXY) on West Texas Intermediate (USOIL). Tables 1, 2, 3 and 4 and Fig. 6 suggest that Bitcoin is the major influence on USOIL prices. There is also an agreement between SVM and MLP models that Ethereum ranks second and the US dollar index (DXY) in third place. However, GRNN model results were the opposite, as the US dollar index comes second and Ethereum in third place.

**Table 1:** SVM results for USOIL

Cryptocurrencies, US dollar and COVID-19	Panel A: DBSVM-C	Panel B: DDSVM-C	Panel C: UBSVM-C	Panel D: UDSVM-C	Panel E: DDSVM + CTC	Panel F: UDSVM + CTC	Panel G: DDSVM + CTNC	Panel H: UDSVM + CTNC
BTCUSD	100	100	100	92.594	100	76.896	23.013	14.055
ETHUSD	59.587	36.814	47.79	100	29.243	64.361	22.283	5.422
DXY	4.113	18.178	9.815	57.977	20.825	47.128	14.024	2.444
Confirmed					74.761	86.608	59.416	13.332
Deaths					66.783	100	50.942	100
Recovered					65.818	86.755	100	11.403
Confirmedn							25.221	1.089
Deathsn							55.578	0.585
Recoveredn							11.523	0.349
*Model parameters*								
Number of points evaluated during search	1121	1120	1121	1101	1174	1111	1109	1080
Minimum error found by search	6.273606	6.011491	5.515289	4.606255	6.508990	1.550218	11.783785	3.290250
Epsilon	0.001	0.001	0.001	0.001	0.001	0.001	0.001	0.001
C	2321.67908	650.878383	29.6388661	5150.4375	34.7919768	127.151128	4215.32855	3143.9348
Gamma	1.35702305	10.9306696	4.49684624	18.8282308	7.12697047	35.7910732	0.4379027	1.34568068
P	0.12406499	0.11687401	0.00673761	0.98599417	0.49706067	0.2071993	0.03877342	1.01122988
Number of support vectors used by the model	52	60	83	162	54	256	61	149
*Analysis of variance*								
Mean target value for input data	58.997368	37.661719	56.387108	48.793622	37.661719	48.793622	37.661719	48.793622
Mean target value for predicted values	58.990804	37.804286	56.683536	48.859386	37.895495	48.801358	37.110248	48.818701
Variance in input data	77.758497	205.40235	25.882874	180.63489	205.40235	180.63489	205.40235	180.63489
Residual	3.1875799	3.1152025	4.7804017	2.7830963	3.4738044	0.6506751	1.6550514	1.5550085
Proportion of variance explained by model (R^2)	0.95901	0.98483	0.81531	0.98459	0.98309	0.99640	0.99194	0.99139
Coefficient of variation (CV)	0.030262	0.046864	0.038775	0.034190	0.049488	0.016532	0.034159	0.025557
Normalized mean square error (NMSE)	0.040993	0.015166	0.184694	0.015407	0.016912	0.003602	0.008058	0.008609
Correlation between actual and predicted	0.979289	0.992619	0.905389	0.992281	0.992191	0.998199	0.996023	0.995711
Maximum error	5.2979261	13.320363	11.977867	9.5037786	12.781792	5.4451013	3.9382116	5.4914609
RMSE	1.7853795	1.7649936	2.186413	1.6682615	1.8638145	0.8066443	1.286488	1.2469998
MSE (Mean Squared Error)	3.1875799	3.1152025	4.7804017	2.7830963	3.4738044	0.6506751	1.6550514	1.5550085
MAE (Mean Absolute Error)	1.1902859	0.4569056	1.1029656	1.0917731	0.9303965	0.497672	0.6937607	0.9644189
MAPE (Mean Absolute Percentage Error)	2.1582771	2082.1471	2.1563491	2.8205657	1999.1466	1.3334963	7.8100362	2.440521

Variables are defined in Appendix 1

**Table 2:** MLP results for USOIL

Cryptocurrencies, US dollar and COVID-19	Panel A: DBMLP-C	Panel B: DDMLP-C	Panel C: UBMLP-C	Panel D: UDMLP-C	Panel E: DDMLP + CTC	Panel F: UDMLP + CTC	Panel G: DDMLP + CTNC	Panel H: UDMLP + CTNC
BTCUSD	100	100	100	45.026	100	17.854	93.293	34.728
ETHUSD	48.63	23.855	39.108	100	17.946	4.489	10.329	5.12
DXY	5.79	3.518	5.353	22.425	6.083	1.884	1.958	7.086
Confirmed					40.083	24.542	86.681	39.864
Deaths					10.14	100	100	100
Recovered					9.123	19.65	71.99	2.762
Confirmedn							2.204	3.704
Deathsn							9.884	27.089
Recoveredn	`						8.923	2.035
*Neural network parameters*								
*Analysis of variance*								
Mean target value for input data	58.997368	37.661719	56.387108	48.793622	37.661719	48.793622	37.369841	48.793622
Mean target value for predicted values	58.956243	37.786673	56.473959	48.685559	37.647716	48.769649	37.416845	48.80453
Variance in input data	77.758497	205.40235	25.882874	180.63489	205.40235	180.63489	203.21038	180.63489
Residual	5.3260727	13.926633	6.3953944	5.3899763	13.917963	4.142072	10.289002	3.4395197
Proportion of variance explained by model (R^2)	0.93150	0.93220	0.75291	0.97016	0.93224	0.97707	0.94937	0.98096
Coefficient of variation (CV)	0.039117	0.099088	0.044849	0.047581	0.099058	0.041711	0.085835	0.038009
Normalized mean square error (NMSE)	0.068495	0.067802	0.247090	0.029839	0.067760	0.022931	0.050632	0.019041
Correlation between actual and predicted	0.965159	0.965678	0.868011	0.985000	0.965539	0.988470	0.974535	0.990492
Maximum error	5.9637008	16.174643	10.438969	10.146544	14.008877	9.1891833	9.7541469	7.9918112
RMSE	2.3078286	3.7318404	2.5289117	2.3216322	3.7306786	2.0352081	3.2076474	1.8545942
MSE (Mean Squared Error)	5.3260727	13.926633	6.3953944	5.3899763	13.917963	4.142072	10.289002	3.4395197
MAE (Mean Absolute Error)	1.8054636	2.6344123	1.7021558	1.6990807	2.9275229	1.38728	2.4526325	1.3531074
MAPE (Mean Absolute Percentage Error)	3.1782505	2534.7846	3.2259454	4.3329987	2197.1943	3.7954588	370.9916	3.4016124

Variables are defined in Appendix 1

**Table 3:** MLP parameters

		Panel A			Panel B			Panel C			Panel D		
Layer	Activation	Neurons	Min. Weight	Max. Weight	Neurons	Min. Weight	Max. Weight	Neurons	Min. Weight	Max. Weight	Neurons	Min. Weight	Max. Weight
Input	Passthru	3			3			3			3		
Hidden 1	Leaky ReLU	12	− 1.512e + 000	1.520e + 000	14	− 1.725e + 000	1.617e + 000	7	− 1.468e + 000	1.105e + 000	16	− 1.709e + 000	1.752e + 000
Output	Linear	1	− 4.750e-001	5.109e-001	1	− 3.708e-001	5.106e-001	1	− 5.817e-001	3.420e-001	1	− 5.739e-001	4.198e-001

		Panel E			Panel F			Panel G			Panel H		
Layer	Activation	Neurons	Min. Weight	Max. Weight	Neurons	Min. Weight	Max. Weight	Neurons	Min. Weight	Max. Weight	Neurons	Min. Weight	Max. Weight
Input	Passthru	6			6			9			9		
Hidden 1	Leaky ReLU	2	− 5.730e-001	5.208e-001	18	− 8.247e-001	9.160e-001	12	− 8.176e-001	9.161e-001	5	− 7.459e-001	9.300e-001
Output	Linear	1	− 2.291e-001	6.013e-001	1	− 3.482e-001	3.840e-001	1	− 4.086e-001	3.320e-001	1	− 1.005e + 000	3.364e-001

Variables are defined in Appendix 1

**Table 4:** GRNN results for USOIL

Cryptocurrencies, US dollar and COVID-19	Panel A: DBGRNN-C		Panel B: DDGRNN-C		Panel C: UBGRNN-C		Panel D: UDGRNN-C	
BTCUSD	100		100		100		99.339	
ETHUSD	0.053		15.116		71.171		100	
DXY	70.79		2.474		0.145		13.631	
Confirmed								
Deaths								
Recovered								
Confirmedn								
Deathsn								
Recoveredn								
Neural network parameters	Evaluations	Error	Evaluations	Error	Evaluations	Error	Evaluations	Error
Starting parameters	1,140	1.0647e + 001	1280	1.4986e + 001	1660	7.6684e + 000	6240	6.3109e + 000
Conjugate gradient	11,571	9.7075e + 000	7040	8.8054e + 000	4482	5.1378e + 000	65,832	4.5139e + 000
*Analysis of variance*								
Mean target value for input data	58.997368		37.661719		56.387108		48.793622	
Mean target value for predicted values	58.92495		37.649087		56.424275		48.817872	
Variance in input data	77.758497		205.40235		25.882874		180.63489	
Residual	7.9530356		2.0652439		1.3681916		2.0577419	
Proportion of variance explained by model (R^2)	0.89772		0.98995		0.94714		0.98861	
Coefficient of variation (CV)	0.047801		0.038158		0.020744		0.029399	
Normalized mean square error (NMSE)	0.102279		0.010055		0.052861		0.011392	
Correlation between actual and predicted	0.948704		0.994981		0.973773		0.994310	
Maximum error	8.3880672		9.6332275		7.6565323		11.12824	
RMSE	2.8201127		1.4370957		1.1696972		1.4344832	
MSE (Mean Squared Error)	7.9530356		2.0652439		1.3681916		2.0577419	
MAE (Mean Absolute Error)	2.2057547		0.6553226		0.5646097		0.7496817	
MAPE (Mean Absolute Percentage Error)	3.8951061		1507.0672		1.0839758		2.2882033	

Cryptocurrencies, US dollar and COVID-19	Panel E: DDGRNN + CTC		Panel F: UDGRNN + CTC		Panel G: DDGRNN + CTNC		Panel H: UDGRNN + CTNC	
BTCUSD	21.845		0.002		0.019		19.39	
ETHUSD	18.633		3.618		77.479		8.794	
DXY	25.646		27.955		68.935		100	
Confirmed	100		52.036		18.554		2.928	
Deaths	87.327		100		100		7.684	
Recovered	84.48		47.498		96.426		21.89	
Confirmedn					0.011		44.318	
Deathsn					0.007		42.021	
Recoveredn					46.24		51.378	
Neural network parameters	Evaluations	Error	Evaluations	Error	Evaluations	Error	Evaluations	Error
Starting parameters	1.280	8.8444e + 000	6240	3.1540e + 000	1,260	2.2364e + 001	6,240	2.9535e + 001
Conjugate gradient	12.992	8.7919e + 000	61,464	8.6368e-001	5,418	1.5696e + 001	19,656	8.4194e + 000
*Analysis of variance*								
Mean target value for input data	37.661719		48.793622		37.661719		48.793622	
Mean target value for predicted values	37.663046		48.793184		37.365807		48.854361	
Variance in input data	205.40235		180.63489		205.40235		180.63489	
Residual	0.091057		0.2496094		0.0681301		4.5101757	
Proportion of variance explained by model (R^2)	0.99956		0.99862		0.99967		0.97503	
Coefficient of variation (CV)	0.008012		0.010239		0.006931		0.043524	
Normalized mean square error (NMSE)	0.000443		0.001382		0.000332		0.024968	
Correlation between actual and predicted	0.999778		0.999310		0.999830		0.987553	
Maximum error	1.015773		1.9821798		1.0647621		12.436363	
RMSE	0.3017566		0.4996092		0.2610174		2.1237174	
MSE (Mean Squared Error)	0.091057		0.2496094		0.0681301		4.5101757	
MAE (Mean Absolute Error)	0.1401054		0.3586232		0.1274421		1.296658	
MAPE (Mean Absolute Percentage Error)	0.6899747		0.9181707		0.266629		3.9252796	

Variables are defined in Appendix 1

#### Scenario 2: Results during downtrend during COVID-19 outbreak

First, when we run the baseline model during the downtrend without adding covid-19 variables, there is an agreement between SVM, MLP and GRNN models on the results of measuring the predictability power of cryptocurrencies and DXY on USOIL. Tables 1, 2, 3 and 4 and Fig. 6 suggest that Bitcoin is the major influence on USOIL prices. Ethereum ranked second, then DXY.

**Fig. 7:**
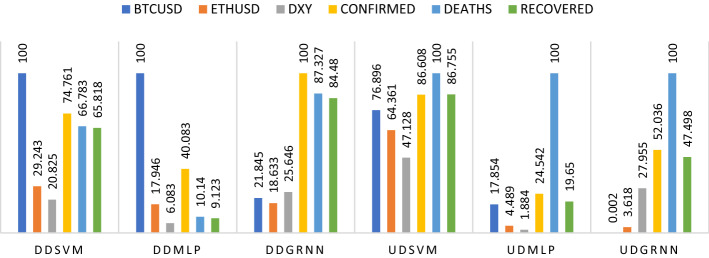
Ranking of cryptocurrencies, DXY and COVID-19 including total cases according to their importance in affecting oil before and during COVID-19. Variables are defined in Appendix 1

Second, we run the model during a downtrend during COVID-19 after adding covid-19 variables (total confirmed, death and recovered cases). From Tables 1,2,3 and 4 and Fig. 7, we find that there is an agreement between SVM and MLP models that Bitcoin is the biggest influence on the price of USOIL, and second are confirmed cases. In third place, the results of SVM refer to recovered cases, while the results of MLP refer to Ethereum. In fourth place, SVM and MLP coincided in the death cases and in the fifth rank, SVM referred to Ethereum, while MLP referred to recovered cases. Then SVM and MLP coincided in the last order of DXY. The results of GRNN were as follows: confirmed cases have the greatest influence, followed by death cases in the second place, and the third place was for cases of recovery, then the dollar in the fourth place, followed by Bitcoin and then Ethereum.

**Fig. 8:**
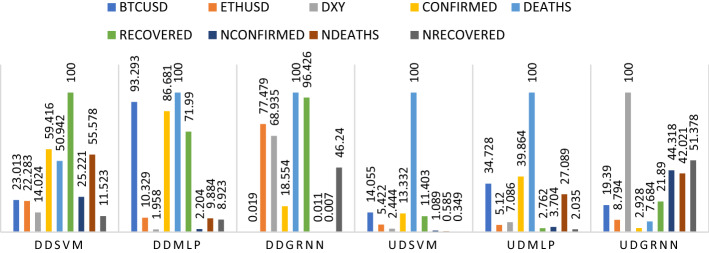
Ranking of cryptocurrencies, DXY and COVID-19 including total and new cases according to their importance in affecting oil before and during COVID-19. Variables are defined in Appendix 1

Third, we run the model during a downtrend during COVID-19 after adding COVID-19 variables (total confirmed, death recovered, new confirmed, new death and new recovered cases). From Tables 1, 2, 3 and 4 and Fig. 8, death cases were the greatest influence according to MLP and GRNN, while recovered cases were the greatest influence according to SVM. The second influence in the order was confirmed cases according to SVM, Bitcoin according to MLP and recovered cases according to GRNN. The third rank was new deaths according to SVM, confirmed cases according to MLP and Ethereum according to GRNN. The fourth rank was death cases according to SVM, recovered cases according to MLP, and DXY according to GRNN. New confirmed cases ranked fifth based on SVM, Ethereum based on MLP, and new recovered cases based on GRNN. Bitcoin ranked sixth based on SVM, new death cases based on MLP, and confirmed cases based on GRNN, Ethereum ranked seventh based on SVM, new recovered cases based on MLP, and bitcoin-based on GRNN, DXY ranked eighth based on SVM and new confirmed cases based on MLP and GRNN, new recovered cases ranked last according to SVM, DXY according to MLP and new death cases according to GRNN.

#### Scenario 3: Results during uptrend before COVID-19 outbreak

When we run the model during the uptrend before COVID-19, there is an agreement between SVM, MLP and GRNN models on the results of measuring the predictability of cryptocurrencies and DXY on USOIL. From Tables 1, 2, 3 and 4 and Fig. 6, it is clear that Bitcoin is the major influence on USOIL prices, Ethereum ranked second, then DXY.

#### Scenario 4: Results during uptrend during COVID-19 outbreak

First, when we run the baseline model during an uptrend during covid-19 without adding COVID-19 variables, there is an agreement between SVM, MLP and GRNN models on the results of measuring the predictability of cryptocurrencies and DXY on USOIL. From Tables 1, 2, 3 and Fig. 6, it is clear that Ethereum is the major influence on USOIL prices, Bitcoin ranked second, then DXY ranked third. We note that there has been an exchange of roles between Bitcoin and Ethereum compared to what happened in the uptrend before COVID-19.

Second, we run the model during an uptrend during COVID-19 after adding COVID-19 variables (total confirmed, death and recovered cases). From Tables 1 and 2 and Fig. 7, we found that there is an agreement among the three models that the greatest influence is death cases, followed by confirmed cases according to MLP and GRNN and recovery cases according to SVM. Then the recovered cases come in third place according to MLP and GRNN and the confirmed cases according to SVM, then Bitcoin in fourth place according to SVM and MLP and DXY according to GRNN. Then the three models agree that Ethereum comes in fifth place and in the end, the least impact is DXY according to SVM and MLP and Bitcoin according to GRNN.

Third, we run the model during an uptrend during COVID-19 after adding covid-19 variables (total confirmed, death recovered, new confirmed, new death and new recovered cases). From Tables 1, 2, 3 and 4 and Fig. 8, death cases were the greatest influence according to SVM and MLP, while DXY were the greatest influence according to GRNN. The second influence in the order was Bitcoin according to SVM, confirmed cases according to MLP and new recovered cases according to GRNN. The third rank was confirmed cases according to SVM, Bitcoin according to MLP and new confirmed cases according to GRNN. The fourth rank was recovered cases according to SVM, and new death cases according to MLP and GRNN. Ethereum ranked fifth based on SVM, DXY based on MLP, and recovered cases based on GRNN. DXY ranked sixth based on SVM, Ethereum based on MLP, and Bitcoin-based on GRNN. New confirmed cases ranked seventh based on SVM and MLP, and Ethereum based on GRNN. New death cases ranked eighth based on SVM, recovered cases based on MLP, and death cases based on GRNN. New recovered cases ranked last according to SVM and MLP and confirmed cases according to GRNN.

#### Scenarios discussion

**Fig. 9:**
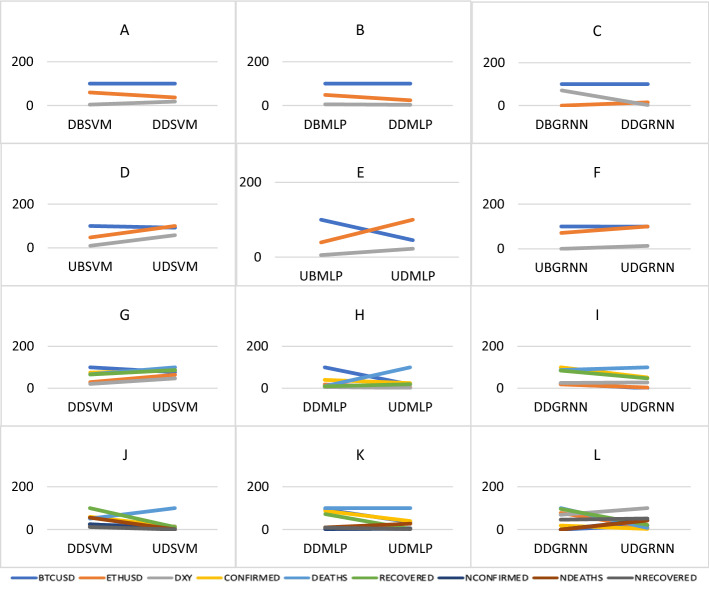
Change in the impact of cryptocurrencies, DXY and the COVID-19 variables during the uptrend and the downtrend before and during COVID-19. Variables are defined in Appendix 1

First, changes in the impact of Bitcoin, Ethereum and DXY during the downtrend before and during COVID-19 are presented in Fig. 9. We observe in Fig. 9 A, B, C that the predictability of Bitcoin did not change according to our three models. While the predictability of Ethereum decreased according to SVM and MLP and increased according to GRNN. The predictability of DXY decreased according to MLP and GRNN and increased according to SVM.

Second, changes in the predictability of Bitcoin, Ethereum and DXY during the uptrend before and during COVID-19 are presented in Fig. 9. We observe in Fig. 9 D, E, F that the predictability of Bitcoin did not change according to GRNN and decreased according to SVM and MLP. The predictability of Ethereum and DXY increased according to all our models.

Third, changes in the impact of Bitcoin, Ethereum, DXY and COVID-19 variables (total confirmed, death and recovered cases) during downtrend and uptrend during COVID-19 are presented in Fig. 9. We observe in Fig. 9 G, H, I that the effect of Bitcoin decreased according to the three models, while the effect of Ethereum increased according to SVM but decreased according to MLP and GRNN. DXY effect increased according to SVM and GRNN but decreased according to MLP, confirmed cases effect decreased according to MLP but increased according to SVM, death cases effect increased according to the three models, recovered cases effect increased according to SVM and MLP but decreased according to GRNN.

Fourth, changes in the impact of Bitcoin, Ethereum, DXY and COVID-19 variables (total confirmed, death recovered, new confirmed, new death and new recovered cases) during down and uptrend during COVID-19. We observe in Fig. 9 J, K, L that the effect of Bitcoin decreased according to SVM and MLP but increased according to GRNN. The effect of Ethereum decreased according to the three models, while the effect of Ethereum increased according to MLP and GRNN but decreased according to SVM. Confirmed cases effect decreased according to the three models, death cases effect decreased according to SVM and GRNN but did not change according to MLP. The recovered cases effect decreased according to the three models, the new confirmed cases effect increased according to MLP and GRNN but decreased according to SVM, new death effect increased according to MLP and GRNN but decreased according to SVM. New recovered cases effect decreased according to SVM and MLP but increased according to GRNN.

Our results can be explained by previous literature that explored the relationship between cryptocurrencies, the US dollar, and oil prices. Musialkowska et al. (2020) conclude that Bitcoin can be considered a weak safe haven. Also, a number of studies (Al-Yahyaee et al., 2019; Charfeddine et al., 2020; Dutta et al., 2020) concluded that cryptocurrencies can be used as a diversifier. In the same line, a strand of research (Al-Yahyaee et al., 2019; Bouri et al., 2017a, b; Das et al., 2020; Dyhrberg, 2016; Guesmi et al., 2019; Selmi et al., 2018) concluded that cryptocurrency can be used in hedging, Therefore, it can be concluded that the reason for the strong predictability of Bitcoin and Ethereum during COVID-19 during the downtrend is due to the fact that individual investors, mutual funds and hedge funds have used cryptocurrencies in hedge, diversification and as a safe haven under conditions of uncertainty during this period. Our results differ from Ji et al. (2019), as they concluded that energy commodities such as oil depend on the dynamics of cryptocurrencies. We have studied the impact of cryptocurrencies and the US dollar as a traditional currency using neural network models to identify the importance of Bitcoin, Ethereum and the dollar, which was presented above. Our results support extant literature (Aloui et al., 2013; Cifarelli & Paladino, 2010; Ferraro et al., 2015; Fratzscher et al., 2014; Jiang & Gu, 2016), as they concluded that there is a relationship between oil prices and the US dollar or exchange rates in which the US dollar is directly or indirectly part, but our results showed the strength of the impact of the US dollar on oil compared to cryptocurrencies before and during the COVID-19 during the uptrend and downtrend.

### Prediction error before and during COVID-19

This section aims to measure forecast error using five metrics, such as Normalized Mean Square Error (NMSE), Root Mean Squared Error (RMSE), Mean Squared Error (MSE), Mean Absolute Error (MAE) and Mean Absolute Percentage Error (MAPE). This helps identify the best prediction model before and during COVID-19 and during the uptrend and downtrend, which will guide policy and decision-makers to the best prediction model when predicting the price of oil under normal conditions and during periods of uncertainty.

**Fig. 10:**
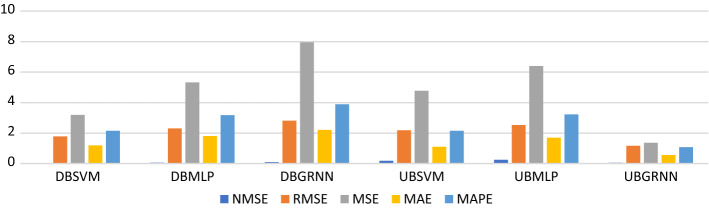
SVM, MLP and GRNN USOIL prediction error before the COVID-19 during uptrend and downtrend. Variables are defined in Appendix 1

First, we measure USOIL prediction error before COVID-19 during the uptrend and downtrend. From Tables 1, 2, 3 and 4 and Fig. 10, we found that the best model to predict USOIL price before COVID-19 during the downtrend is SVM, since its prediction error is less than MLP and GRNN, and the best model to forecast before COVID-19 during the uptrend is GRNN because its prediction error is less than SVM and MLP.

**Fig. 11:**
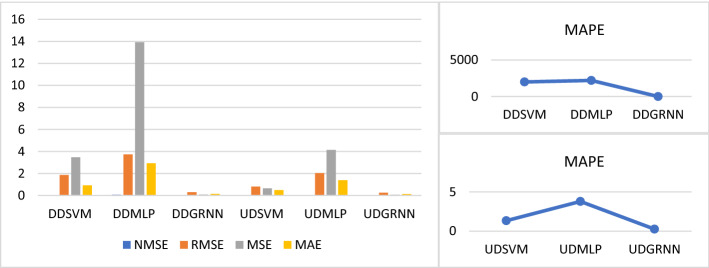
SVM, MLP and GRNN USOIL prediction error during COVID-19 including total cases during uptrend and downtrend. Variables are defined in Appendix 1

Second, we measure USOIL prediction error during COVID-19 during uptrend and downtrend after adding COVID-19 variables (total confirmed, death and recovered cases). From Tables 1, 2, 3 and 4 and Fig. 11, we found that the best prediction model during the uptrend and downtrend is GRNN because the prediction error is less than SVM and MLP prediction error. Thus, we recommend using GRNN when predicting the price of USOIL during periods of uncertainty similar to COVID-19 whether the market trend is optimistic or downtrend.

**Fig. 12:**
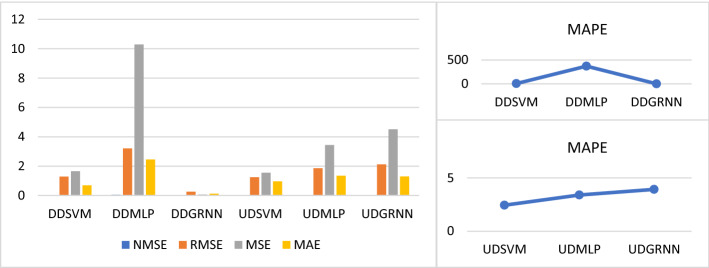
SVM, MLP and GRNN USOIL prediction error during COVID-19 including total and new cases during uptrend and downtrend. Variables are defined in Appendix 1

Third, we calculate USOIL prediction error during COVID-19 during uptrend and downtrend after adding COVID-19 variables (total confirmed, death recovered, new confirmed, new death and new recovered cases). From Tables 1, 2, 3 and 4 and Fig. 12. We found that the best model to predict USOIL price during COVID-19 during the downtrend is GRNN, since its prediction error is less than SVM and MLP, and the best model to forecast during COVID-19 during the uptrend is SVM because its prediction error is less than MLP and GRNN.

We observe from the second and third that the best model to forecast the downtrend during COVID-19 with the use of the uncertainty source data as a total in addition to the change in it is GRNN. As for the best model during the uptrend during COVID-19, if we use the data from the source of uncertainty as a total is GRNN, but if we use the data as a total. In addition to changing it, the best model will become SVM, and this is what we advise the policy and decision-makers when forecasting the price of USOIL during periods of uncertainty.

### The role of COVID-19 variables in improving the ability of models to predict the downtrend during the COVID-19

**Fig. 13:**
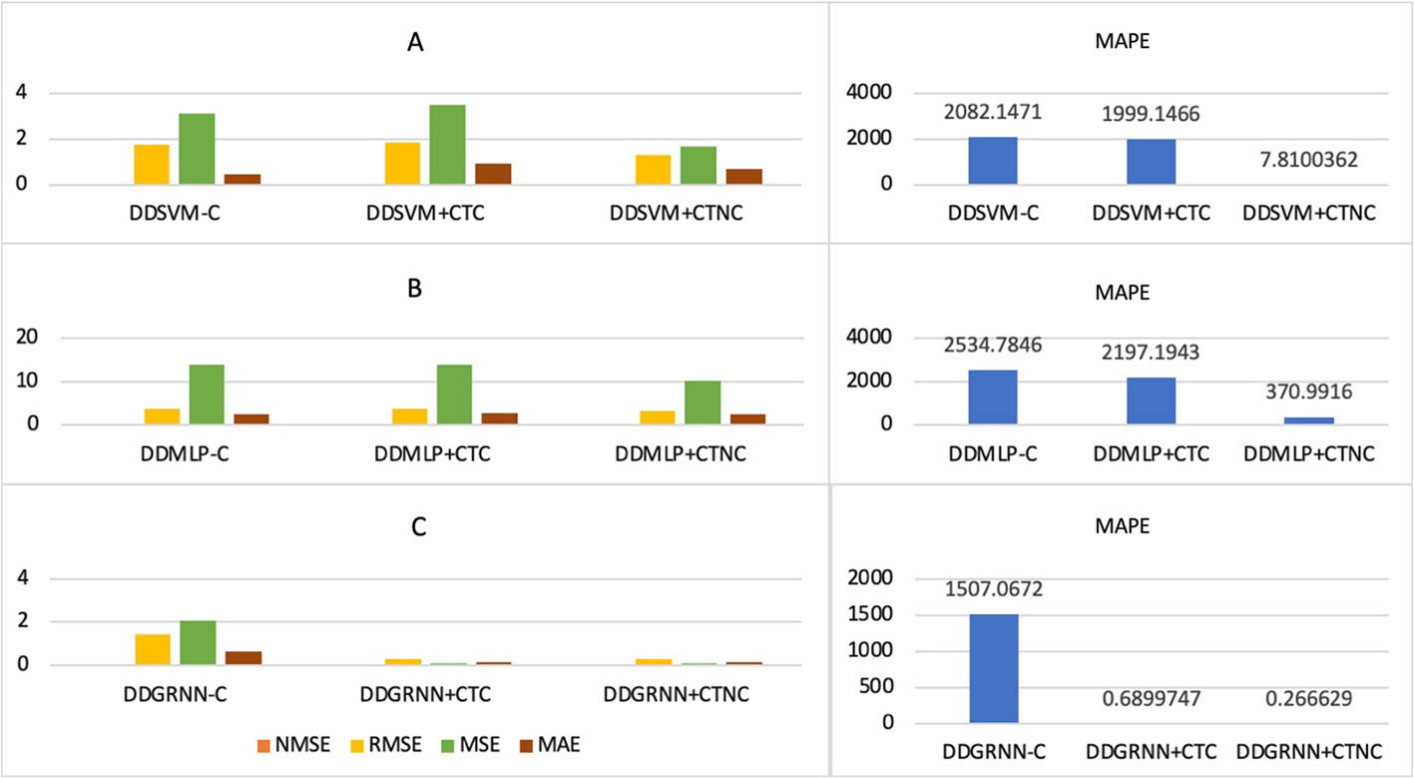
The role of COVID-19 variables in improving the ability of models to predict the downtrend during COVID-19. Variables are defined in Appendix 1

Tables 1, 2, 3 and 4 and Fig. 13 present our results when we run the baseline model during a downtrend without adding covid-19 variables. The prediction error shown in Fig. 13 A was greater than the prediction error when we run the model during a downtrend during COVID-19 after adding COVID-19 variables (total confirmed, death and recovered cases) as shown in Fig. 13 B which was greater than the prediction error when we run the model during downtrend during COVID-19 after adding covid-19 variables (new confirmed, new death and new recovered cases) as shown in Fig. 13 C. Therefore, we recommend, during periods of uncertainty, to use the source of uncertainty data as a total, in addition to the new change in it, as this will improve the ability of the models used to predict the price of USOIL by reducing the prediction error.

### Robustness analysis

To ensure the accuracy of our results, we performed the analysis using Brent crude oil (UKOIL) instead of USOIL, and the results were very similar, as shown in Tables 5, 6, 7 and 8.

**Table 5:** SVM results for UKOIL

Cryptocurrencies, US dollar and COVID-19	Panel A: DBSVM-C	Panel B: DDSVM-C	Panel C: UBSVM-C	Panel D: UDSVM-C	Panel E: DDSVM + CTC	Panel F: UDSVM + CTC	Panel G: DDSVM + CTNC	Panel H: UDSVM + CTNC
BTCUSD	100	100	100	100	100	44.136	100	45.689
ETHUSD	53.149	19.899	59.026	95.416	62.636	4.605	18.324	29.554
DXY	5.932	3.99	13.37	69.112	65.129	2.26	16.31	10.756
Confirmed					70.084	45.645	29.65	12.421
Deaths					56.949	100	20.063	100
Recovered					71.848	11.099	19.968	12.604
Confirmedn							21.46	2.772
Deathsn							11.928	0.924
Recoveredn							4.018	0.894
*Model parameters*								
Number of points evaluated during search	1180	1087	1116	1120	1104	1103	1135	1125
Minimum error found by search	6.438740	7.735254	6.839954	3.827987	4.301241	1.799730	5.964681	2.764625
Epsilon	0.001	0.001	0.001	0.001	0.001	0.001	0.001	0.001
C	822.710934	412.005647	37.9697847	50.5133233	693.539358	16,638.5191	484.229896	527.879822
Gamma	1.26340968	4.20913374	4.54361404	48.8899096	13.7676343	1.35732445	1.35720881	2.31150422
P	0.94235807	0.2193308	0.056446	0.77364565	0.13919743	0.00205604	1.10581726	0.36633093
Number of support vectors used by the model	42	65	78	189	61	312	39	231
*Analysis of variance*								
Mean target value for input data	68.45193	45.297973	64.975542	51.639263	45.297973	51.639263	45.297973	51.639263
Mean target value for predicted values	68.754852	45.406904	65.186552	51.832769	42.598705	51.783209	42.450619	51.658662
Variance in input data	84.699054	223.16335	23.074837	176.37446	223.16335	176.37446	223.16335	176.37446
Residual (unexplained) variance after model fit	3.8451233	3.7987169	5.6586021	2.4383013	0.0255208	1.3204774	1.4198372	1.2433042
Proportion of variance explained by model (R^2)	0.95460	0.98298	0.75477	0.98618	0.99989	0.99251	0.99364	0.99295
Coefficient of variation (CV)	0.028646	0.043027	0.036610	0.030239	0.003527	0.022253	0.026305	0.021593
Normalized mean square error (NMSE)	0.045397	0.017022	0.245228	0.013825	0.000114	0.007487	0.006362	0.007049
Correlation between actual and predicted	0.977612	0.991525	0.870701	0.993203	0.999926	0.996313	0.995795	0.996473
Maximum error	7.1741524	7.0145767	12.870142	7.7163957	0.842902	4.9764529	4.1634232	4.4844113
RMSE (Root Mean Squared Error)	1.9608986	1.9490297	2.3787816	1.5615061	0.1597523	1.1491203	1.1915692	1.1150355
MSE (Mean Squared Error)	3.8451233	3.7987169	5.6586021	2.4383013	0.0255208	1.3204774	1.4198372	1.2433042
MAE (Mean Absolute Error)	1.4777346	1.1705749	1.1942757	1.0837883	0.1438743	0.7524677	1.0418887	0.7813221
MAPE (Mean Absolute Percentage Error)	2.2870992	3.0749344	1.9902625	2.5737761	0.3731476	1.681572	2.6793774	1.7407687

Variables are defined in Appendix 1

**Table 6:** MLP results for UKOIL

Cryptocurrencies, US dollar and COVID-19	Panel A: DBMLP-C	Panel B: DDMLP-C	Panel C: UBMLP-C	Panel D: UDMLP-C	Panel E: DDMLP + CTC	Panel F: UDMLP + CTC	Panel G: DDMLP + CTNC	Panel H: UDMLP + CTNC
BTCUSD	100	100	100	100	67.175	19.246	100	26.912
ETHUSD	41.203	34.175	24.147	41.538	23.401	4.245	14.714	11.314
DXY	6.155	4.566	3.671	12.993	7.719	1.958	5.875	25.838
Confirmed					43.734	22.629	12.119	12.458
Deaths					100	100	4.156	100
Recovered					65.91	18.017	5.812	59.763
Confirmedn							1.21	1.867
Deathsn							2.005	1.266
Recoveredn	`						2.482	0.218
Neural network parameters								
*Analysis of variance*								
Mean target value for input data	68.45193	45.297973	64.975542	51.639263	42.604769	51.639263	42.292031	51.639263
Mean target value for predicted values	68.595025	45.428399	65.147022	51.548305	42.655061	51.645017	42.2945	51.575385
Variance in input data	84.699054	223.16335	23.074837	176.37446	194.4023	176.37446	191.0825	176.37446
Residual (unexplained) variance after model fit	5.8914355	6.8975242	7.3653084	4.358057	3.6560543	2.9667855	12.781874	3.6260698
Proportion of variance explained by model (R^2)	0.035459	0.96909	0.68081	0.97529	0.98119	0.98318	0.93311	0.97944
Coefficient of variation (CV)	0.039117	0.057979	0.041768	0.040427	0.044880	0.033355	0.084535	0.036876
Normalized mean square error (NMSE)	0.069557	0.030908	0.319192	0.024709	0.018807	0.016821	0.066892	0.020559
Correlation between actual and predicted	0.964744	0.984468	0.827534	0.987592	0.990580	0.991565	0.966221	0.989679
Maximum error	5.0114243	7.2651445	11.644311	7.3481323	3.9254817	6.4197284	10.294254	7.0417063
RMSE (Root Mean Squared Error)	2.4272279	2.6263138	2.7139102	2.087596	1.9120811	1.7224359	3.5751747	1.9042242
MSE (Mean Squared Error)	5.8914355	6.8975242	7.3653084	4.358057	3.6560543	2.9667855	12.781874	3.6260698
MAE (Mean Absolute Error)	1.9976056	2.0170177	1.796905	1.6112244	1.5046992	1.2684834	2.8221063	1.4438432
MAPE (Mean Absolute Percentage Error)	3.0029124	5.5672779	2.9274051	3.5506671	3.7639619	2.9120625	7.3767093	3.3091786

Variables are defined in Appendix 1

**Table 7:** MLP parameters

		Panel A			Panel B			Panel C			Panel D		
Layer	Activation	Neurons	Min. Weight	Max. Weight	Neurons	Min. Weight	Max. Weight	Neurons	Min. Weight	Max. Weight	Neurons	Min. Weight	Max. Weight
Input	Passthru	3			3			3			3		
Hidden 1	Leaky ReLU	17	− 1.657e + 000	1.808e + 000	14	− 1.734e + 000	1.672e + 000	8	− 1.181e + 000	1.512e + 000	18	− 1.786e + 000	1.749e + 000
Output	Linear	1	− 2.149e-001	3.594e-001	1	− 5.668e-001	6.167e-001	1	− 3.134e-001	4.916e-001	1	− 4.213e-001	3.469e-001

		Panel E			Panel F			Panel G			Panel H		
Layer	Activation	Neurons	Min. Weight	Max. Weight	Neurons	Min. Weight	Max. Weight	Neurons	Min. Weight	Max. Weight	Neurons	Min. Weight	Max. Weight
Input	Passthru	6			6			9			9		
Hidden 1	Leaky ReLU	16	− 9.562e-001	8.562e-001	18	− 8.262e-001	9.245e-001	7	− 8.341e-001	7.086e-001	19	− 9.480e-001	9.361e-001
Output	Linear	1	− 2.843e-001	6.084e-001	1	− 3.047e-001	3.807e-001	1	− 3.698e-001	5.213e-001	1	− 5.755e-001	7.551e-001

Variables are defined in Appendix 1

**Table 8:** GRNN results for UKOIL

Cryptocurrencies, US dollar and COVID-19	Panel A: DBGRNN-C		Panel B: DDGRNN-C		Panel C: UBGRNN-C		Panel D: UDGRNN-C	
BTCUSD	100		100		100		100	
ETHUSD	0.042		19.077		11.623		96.096	
DXY	65.095		0.903		4.608		11.454	
Confirmed								
Deaths								
Recovered								
Confirmedn								
Deathsn								
Recoveredn								
Neural network parameters	Evaluations	Error	Evaluations	Error	Evaluations	Error	Evaluations	Error
Starting parameters	1,140	1.0453e + 001	1,480	9.6367e + 000	1,660	8.7045e + 000	6,240	4.2277e + 000
Conjugate gradient	8,835	9.5219e + 000	6,512	6.6846e + 000	5,229	8.7045e + 000	55,536	3.2590e + 000
*Analysis of variance*								
Mean target value for input data	68.45193		45.297973		64.975542		51.639263	
Mean target value for predicted values	68.390703		45.333859		65.010855		51.659477	
Variance in input data	84.699054		223.16335		23.074837		176.37446	
Residual (unexplained) variance after model fit	7.6736421		1.363231		3.6942362		1.4983845	
Proportion of variance explained by model (R^2)	0.90940		0.99389		0.83990		0.99150	
Coefficient of variation (CV)	0.040468		0.025775		0.029581		0.023705	
Normalized mean square error (NMSE)	0.090599		0.006109		0.160098		0.008495	
Correlation between actual and predicted	0.954598		0.996954		0.919222		0.995763	
Maximum error	8.8308393		4.1746186		11.5207		8.2193331	
RMSE (Root Mean Squared Error)	2.7701339		1.1675748		1.9220396		1.2240852	
MSE (Mean Squared Error)	7.6736421		1.363231		3.6942362		1.4983845	
MAE (Mean Absolute Error)	2.1537756		0.7596655		1.0241735		0.7564913	
MAPE (Mean Absolute Percentage Error)	3.2495397		2.1533773		1.695067		1.8750164	

Cryptocurrencies, US dollar and COVID-19	Panel E: DDGRNN + CTC		Panel F: UDGRNN + CTC		Panel G: DDGRNN + CTNC		Panel H: UDGRNN + CTNC	
BTCUSD	91.944		0		100		100	
ETHUSD	54.444		3.427		17.332		4.733	
DXY	75.846		20.595		3.693		4.329	
Confirmed	80.174		49.675		0.29		0.03	
Deaths	69.525		100		18.742		0.159	
Recovered	100		70.381		12.626		2.533	
Confirmedn					4.871		0.103	
Deathsn					4.869		1.544	
Recoveredn					5.016		1.38	
Neural network parameters	Evaluations	Error	Evaluations	Error	Evaluations	Error	Evaluations	Error
Starting parameters	1300	4.9822e + 000	6240	2.1528e + 000	1280	9.5603e + 000	6240	2.8267e + 001
Conjugate gradient	10,335	4.7031e + 000	47,736	8.2358e-001	7744	4.8939e + 000	30,576	1.2425e + 001
*Analysis of variance*								
Mean target value for input data	45.297973		51.639263		45.297973		51.639263	
Mean target value for predicted values	42.60542		51.637701		42.291837		51.709664	
Variance in input data	223.16335		176.37446		223.16335		176.37446	
Residual (unexplained) variance after model fit	0.049885		0.2786358		0.0981458		2.2343994	
Proportion of variance explained by model (R^2)	0.99978		0.99842		0.99956		0.98733	
Coefficient of variation (CV)	0.004931		0.010222		0.006916		0.028947	
Normalized mean square error (NMSE)	0.000224		0.001580		0.000440		0.012668	
Correlation between actual and predicted	0.999854		0.999211		0.999703		0.993766	
Maximum error	0.8513998		2.2581521		1.1225908		8.774853	
RMSE (Root Mean Squared Error)	0.2233496		0.5278597		0.3132824		1.4947907	
MSE (Mean Squared Error)	0.049885		0.2786358		0.0981458		2.2343994	
MAE (Mean Absolute Error)	0.1140406		0.3792303		0.1991017		1.0168639	
MAPE (Mean Absolute Percentage Error)	0.2199081		0.8419498		0.4132256		2.4257221	

Variables are defined in Appendix 1

## Summary and conclusion

The global COVID-19 pandemic has created massive losses and instabilities in global markets (Gradojevic & Kukolj, 2022; Jana & Ghosh, 2022; Jana et al., 2022; Kapoor et al., 2021), especially in oil markets. Thus, building an accurate model to predict oil prices during the bear and bull oil market can offer investors and policymakers the knowledge to take correct decisions in escaping crashes. Our study contributes to the current research by proposing advanced machine learning models to explore the role of cryptocurrencies and the US dollar in predicting oil prices pre and during the COVID-19 pandemic. In this paper, the effect of COVID-19 and two leading cryptocurrencies on the efficiency of predicting oil prices is analyzed via the application of three neural network models (i.e., SVM, MLP and GRNN) over a long period from January 1, 2018, to July 5, 2021, to decrease the bias and the misspecification errors produced by the parametric models.

Our results indicate that Bitcoin is the most influential in predicting oil prices during the bear and bull oil market before COVID-19 and during the downtrend during COVID-19. Ethereum has become the most influential during the bull oil market during COVID-19. The reason for this may be due to Tesla cancelling dealing in Bitcoin and the statement of its chairman that the reason for this is the use of fossil fuels in mining. In addition, Bitcoin has been banned in China during this period. After adding COVID-19 variables to our model, we found that they became more important than Ethereum and the US dollar index during the downtrend, and Bitcoin continued to be the most influential according to SVM and MLP, while COVID-19 variables became the most influential during the uptrend, and the most influential variable was death cases according to the three models. Our results also suggest that the most accurate model to predict the price of oil under the conditions of uncertainty that prevailed in the world during the downtrend during COVID-19 is GRNN and during the uptrend also if the COVID-19 data is used as a total case alone, but if we add the new cases, the most accurate model is SVM. Though the best prediction model under normal conditions before COVID-19 during an uptrend is SVM and during a downtrend is GRNN. Likewise, our results demonstrate that COVID-19 variables are a very rich source of information for predicting the volatility of oil prices, and the inclusion of COVID-19 variables in our models showed consistent outperformance. Consequently, governments may consider the role of COVID-19 variables in formulating policy procedures to attenuate the turmoil and uncertainty of the crude oil markets.

Our results have several policy implications for investors, policymakers and regulators. Policymakers and investors should consider the market condition when predicting oil prices (bull market vs. bear market). This is more predominant during major uncertainties and outbreaks such as COVID-19. Second, our results confirm the role of cryptocurrencies in predicting oil prices during bear and bull oil market conditions. This result may assist investors and policymakers construct more accurate prediction models based on different market states. Third, our results during extreme conditions (bear and bull) suggest a high-value prospect for investors to mix cryptocurrencies and crude oil prices for portfolio hedging and trading strategies during the COVID-19 outbreak. Finally, future research may include more predictive macroeconomic variables to reduce prediction errors. Also, future research may explore the applicability of this study to other markets, such as stock and gold markets.

## Data Availability

Data available on request from the authors.

## Appendix 1: Variable definitions

**Table:**

Abbreviation	Definition
BTCUSD	Bitcoin exchange rate in US dollars
ETHUSD	Ethereum exchange rate in US dollars
DXY	US dollar currency index
USOIL	Price of West Texas Intermediate crude oil barrel in US dollars
UKOIL	Price of Brent crude oil barrel in US dollars
CONFIRMED	The total number of confirmed cases of the COVID-19 worldwide
DEATHS	The total number of COVID-19 death cases worldwide
RECOVRERED	The total number of COVID-19 recovered cases worldwide
NCONFIRMED	The daily number of new confirmed cases of the COVID-19 worldwide
NRECOVRERED	The daily number of new COVID-19 recovered cases worldwide
NDEATHS	The daily number of new COVID-19 death cases worldwide
DBxxx	The results of running xxx model during the downtrend before Covid-19
DDxxx	The results of running xxx model during the downtrend trend during Covid-19
UBxxx	The results of running xxx model during the uptrend before Covid-19
UDxxx	The results of running xxx model during the uptrend during Covid-19
xxxxx-C	The results of running xxx model without adding COVID-19 data
xxxxx + CTC	The results of running xxx model after adding COVID-19 data as a total cases
xxxxx + CTNC	The results of running xxx model after adding COVID-19 data as a total and new cases

## Footnotes

1 See https://coinmarketcap.com

2 See https://www.tradingview.com

3 See https://coinmarketcap.com

## References

[CR3] Abdou HA (2009). Credit scoring models for Egyptian banks: neural nets and genetic programming versus conventional techniques.

[CR4] Abdou HA, Ellelly NN, Elamer AA, Hussainey K, Yazdifar H (2021). Corporate governance and earnings management nexus: Evidence from the UK and Egypt using neural networks. International Journal of Finance & Economics.

[CR5] Abdou HA, Mitra S, Fry J, Elamer AA (2019). Would two-stage scoring models alleviate bank exposure to bad debt?. Expert Systems with Applications.

[CR6] Abdou HA, Pointon J, El-Masry A, Olugbode M, Lister RJ (2012). A variable impact neural network analysis of dividend policies and share prices of transportation and related companies. Journal of International Financial Markets, Institutions and Money.

[CR7] Abedin M Z, Moon M H, Kabir Hassan M, Hajek Petr (2021). Deep learning-based exchange rate prediction during the COVID-19 pandemic. Annals of Operations Research.

[CR8] Akram QF (2009). Commodity prices, interest rates and the dollar. Energy Economics.

[CR9] Albitar K, Al-Shaer H, Elmarzouky M (2021). Do assurance and assurance providers enhance COVID-related disclosures in CSR reports? An examination in the UK context. International Journal of Accounting and Information Management.

[CR10] Albulescu CT, Ajmi AN (2021). Oil price and US dollar exchange rate: Change detection of bi-directional causal impact. Energy Economics.

[CR11] Aloui R, Ben Aïssa MS, Nguyen DK (2013). Conditional dependence structure between oil prices and exchange rates: A copula-GARCH approach. Journal of International Money and Finance.

[CR12] Alshater MM, Kampouris I, Marashdeh H, Atayah OF, Banna H (2022). Early warning system to predict energy prices: The role of artificial intelligence and machine learning. Annals of Operations Research.

[CR13] Al-Yahyaee KH, Mensi W, Al-Jarrah IMW, Hamdi A, Kang SH (2019). Volatility forecasting, downside risk, and diversification benefits of Bitcoin and oil and international commodity markets: A comparative analysis with yellow metal. North American Journal of Economics and Finance.

[CR14] Bašta M, Molnár P (2018). Oil market volatility and stock market volatility. Finance Research Letters.

[CR15] Baur DG, Dimpfl T, Kuck K (2018). Bitcoin, gold and the US dollar – A replication and extension. Finance Research Letters.

[CR16] Bénassy-Quéré A, Mignon V, Penot A (2007). China and the relationship between the oil price and the dollar. Energy Policy.

[CR17] Blomberg SB, Harris ES (1995). The commodity-consumer price connection: Fact or Fable?. Economic Policy Review.

[CR18] Bouri E, Gupta R, Tiwari AK, Roubaud D (2017). Does Bitcoin hedge global uncertainty? Evidence from wavelet-based quantile-in-quantile regressions. Finance Research Letters.

[CR19] Bouri E, Jalkh N, Molnár P, Roubaud D (2017). Bitcoin for energy commodities before and after the December 2013 crash: Diversifier, hedge or safe haven?. Applied Economics.

[CR20] Charfeddine L, Benlagha N, Maouchi Y (2020). Investigating the dynamic relationship between cryptocurrencies and conventional assets: Implications for financial investors. Economic Modelling.

[CR21] Chen J, Lim CP, Tan KH, Govindan K, Kumar A (2021). Artificial intelligence-based human-centric decision support framework: An application to predictive maintenance in asset management under pandemic environments. Annals of Operations Research.

[CR22] Cifarelli G, Paladino G (2010). Oil price dynamics and speculation a multivariate financial approach. Energy Economics.

[CR23] Corbet S, Lucey B, Urquhart A, Yarovaya L (2019). Cryptocurrencies as a financial asset: A systematic analysis. International Review of Financial Analysis.

[CR24] Coudert V, Mignon V, Penot A (2007). Oil price and the dollar. Energy Studies Review.

[CR25] Darby MR (1982). The price of oil and world inflation and recession. The American Economic Review.

[CR26] Das D, Le Roux CL, Jana RK, Dutta A (2020). Does Bitcoin hedge crude oil implied volatility and structural shocks? A comparison with gold, commodity and the US Dollar. Finance Research Letters.

[CR27] Ding L, Vo M (2012). Exchange rates and oil prices: A multivariate stochastic volatility analysis. Quarterly Review of Economics and Finance.

[CR28] DTREG (2021) DTREG: Predictive Modeling Software. User manual. available at: https://www.dtreg.com/uploaded/downloadfile/DownloadFile_5.pdf

[CR29] Dutta A, Das D, Jana RK, Vo XV (2020). COVID-19 and oil market crash: Revisiting the safe haven property of gold and Bitcoin. Resources Policy.

[CR30] Dyhrberg AH (2016). Hedging capabilities of bitcoin. Is it the virtual gold?. Finance Research Letters.

[CR31] Elmarzouky M, Albitar K, Hussainey K (2021). Covid-19 and performance disclosure: Does governance matter?. International Journal of Accounting and Information Management.

[CR32] Ferraro D, Rogoff K, Rossi B (2015). Can oil prices forecast exchange rates? An empirical analysis of the relationship between commodity prices and exchange rates. Journal of International Money and Finance.

[CR33] Fratzscher M, Schneider D, Van Robays I (2014). Oil prices, exchange rates and asset prices. SSRN Electronic Journal.

[CR34] Ftiti Z, Louhichi W, Ameur H B (2021). Cryptocurrency volatility forecasting: What can we learn from the first wave of the COVID-19 outbreak?. Annals of Operations Research.

[CR35] Gajardo G, Kristjanpoller WD, Minutolo M (2018). Does Bitcoin exhibit the same asymmetric multifractal cross-correlations with crude oil, gold and DJIA as the Euro, Great British Pound and Yen?. Chaos, Solitons and Fractals.

[CR36] Gallersdörfer U, Klaaßen L, Stoll C (2020). Energy Consumption of Cryptocurrencies Beyond Bitcoin. Joule.

[CR37] Ghazani MM, Khosravi R (2020). Multifractal detrended cross-correlation analysis on benchmark cryptocurrencies and crude oil prices. Physica a: Statistical Mechanics and Its Applications.

[CR38] Gradojevic N, Kukolj D (2022). Unlocking the black box: Non-parametric option pricing before and during COVID-19. Annals of Operations Research.

[CR39] Guesmi K, Saadi S, Abid I, Ftiti Z (2019). Portfolio diversification with virtual currency: Evidence from bitcoin. International Review of Financial Analysis.

[CR40] Hayes AS (2017). Cryptocurrency value formation: An empirical study leading to a cost of production model for valuing bitcoin. Telematics and Informatics.

[CR41] Houcine B, Zouheyr G, Abdessalam B, Youcef H, Hanane A (2020). The relationship between crude oil prices, EUR/USD exchange rate and gold prices. International Journal of Energy Economics and Policy.

[CR42] Huynh, T. L. D., Shahbaz, M., Nasir, M. A., & Ullah, S. (2020). Financial modelling, risk management of energy instruments and the role of cryptocurrencies. *Annals of Operations Research*, *0123456789*.

[CR43] Jana RK, Ghosh I (2022). A residual driven ensemble machine learning approach for forecasting natural gas prices: Analyses for pre-and during-COVID-19 phases. Annals of Operations Research.

[CR44] Jana RK, Ghosh I, Jawadi F, Uddin GS, Sousa RM (2022). COVID-19 news and the US equity market interactions: An inspection through econometric and machine learning lens. Annals of Operations Research.

[CR45] Jareño F, González MDLO, López R, Ramos AR (2021). Cryptocurrencies and oil price shocks: A NARDL analysis in the COVID-19 pandemic. Resources Policy.

[CR46] Ji Q, Bouri E, Lau CKM, Roubaud D (2019). Dynamic connectedness and integration in cryptocurrency markets. International Review of Financial Analysis.

[CR47] Jiang J, Gu R (2016). Asymmetrical long-run dependence between oil price and US dollar exchange rate - Based on structural oil shocks. Physica a: Statistical Mechanics and Its Applications.

[CR48] Kapoor K, Bigdeli AZ, Dwivedi YK, Raman R (2021). How is COVID-19 altering the manufacturing landscape? A literature review of imminent challenges and management interventions. Annals of Operations Research.

[CR49] Karim AE, Albitar K, Elmarzouky M (2021). A novel measure of corporate carbon emission disclosure, the effect of capital expenditures and corporate governance. Journal of Environmental Management.

[CR50] Kazancoglu I, Ozbiltekin-Pala M, Mangla SK, Kumar A, Kazancoglu Y (2022). Using emerging technologies to improve the sustainability and resilience of supply chains in a fuzzy environment in the context of COVID-19. Annals of Operations Research.

[CR51] Khalilpourazari S, Hashemi Doulabi H (2022). Designing a hybrid reinforcement learning based algorithm with application in prediction of the COVID-19 pandemic in Quebec. Annals of Operations Research.

[CR52] Kim HM, Bock GW, Lee G (2021). Predicting Ethereum prices with machine learning based on Blockchain information. Expert Systems with Applications.

[CR53] Klein T, Pham Thu H, Walther T (2018). Bitcoin is not the New Gold – A comparison of volatility, correlation, and portfolio performance. International Review of Financial Analysis.

[CR54] Kristjanpoller W, Minutolo MC (2021). Asymmetric multi-fractal cross-correlations of the price of electricity in the US with crude oil and the natural gas. Physica a: Statistical Mechanics and Its Applications.

[CR55] Krugman, P. R., & Obstfeld, M. (2003). International economics: theory and policy. *The Addison-Wesley Series in Economics*.

[CR56] Kumar P, Singh RK, Shahgholian A (2022). Learnings from COVID-19 for managing humanitarian supply chains: Systematic literature review and future research directions. Annals of Operations Research.

[CR57] Kumar S, Sharma D, Rao S, Lim WM, Mangla SK (2022). Past, present, and future of sustainable finance: Insights from big data analytics through machine learning of scholarly research. Annals of Operations Research.

[CR58] Li J, Li N, Peng J, Cui H, Wu Z (2019). Energy consumption of cryptocurrency mining: A study of electricity consumption in mining cryptocurrencies. Energy.

[CR59] Lin FL, Chen YF, Yang SY (2016). Does the value of US dollar matter with the price of oil and gold? A dynamic analysis from time-frequency space. International Review of Economics and Finance.

[CR61] Lizardo RA, Mollick AV (2010). Oil price fluctuations and U. S. dollar exchange rates. Energy Economics.

[CR62] Mensi W, Sensoy A, Vo XV, Kang SH (2020). Impact of COVID-19 outbreak on asymmetric multifractality of gold and oil prices. Resources Policy.

[CR63] Mo B, Nie H, Jiang Y (2018). Dynamic linkages among the gold market, US dollar and crude oil market. Physica A: Statistical Mechanics and Its Applications.

[CR64] Murphy, J. J. (1999). Technical analysis of the financial markets: A comprehensive guide to trading methods and applications. Penguin.

[CR65] Musialkowska I, Kliber A, Świerczyńska K, Marszałek P (2020). Looking for a safe-haven in a crisis-driven Venezuela: The Caracas stock exchange vs gold, oil and bitcoin. Transforming Government: People, Process and Policy.

[CR66] Nyawa S, Tchuente D, Fosso-Wamba S (2022). COVID-19 vaccine hesitancy: A social media analysis using deep learning. Annals of Operations Research.

[CR67] O’Dwyert KJ, Malone D (2014). Bitcoin mining and its energy footprint. IET Conference Publications.

[CR68] Okorie DI, Lin B (2020). Crude oil price and cryptocurrencies: Evidence of volatility connectedness and hedging strategy. Energy Economics.

[CR69] Palombizio E, Morris I (2012). Forecasting exchange rates using leading economic indicators. Open Access Scientific Reports.

[CR70] Park BJ (2022). The COVID-19 pandemic, volatility, and trading behavior in the bitcoin futures market. Research in International Business and Finance.

[CR71] Queiroz MM, Fosso Wamba S (2021). A structured literature review on the interplay between emerging technologies and COVID-19 – insights and directions to operations fields. Annals of Operations Research.

[CR72] Queiroz MM, Ivanov D, Dolgui A, Fosso Wamba S (2020). Impacts of epidemic outbreaks on supply chains: Mapping a research agenda amid the COVID-19 pandemic through a structured literature review. Annals of Operations Research.

[CR73] Rosenblatt F (1958). The perceptron: A probabilistic model for information storage and organization in the brain. Psychological Review.

[CR75] Sadorsky P (2000). The empirical relationship between energy futures prices and exchange rates. Energy Economics.

[CR76] Salisu AA, Vo XV, Lawal A (2021). Hedging oil price risk with gold during COVID-19 pandemic. Resources Policy.

[CR77] Selmi R, Mensi W, Hammoudeh S, Bouoiyour J (2018). Is Bitcoin a hedge, a safe haven or a diversifier for oil price movements? A comparison with gold. Energy Economics.

[CR78] Smales LA (2019). Bitcoin as a safe haven: Is it even worth considering?. Finance Research Letters.

[CR79] Turhan I, Hacihasanoglu E, Soytas U (2013). Oil prices and emerging market exchange rates. Emerging Markets Finance and Trade.

[CR80] Wen F, Xiao J, Huang C, Xia X (2018). Interaction between oil and US dollar exchange rate: Nonlinear causality, time-varying influence and structural breaks in volatility. Applied Economics.

[CR81] Van Wijk, D. (2013). What can be expected from the BitCoin. *Erasmus Universiteit Rotterdam*, *18*.

[CR82] Wu CC, Chung H, Chang YH (2012). The economic value of co-movement between oil price and exchange rate using copula-based GARCH models. Energy Economics.

[CR83] Yousefi A, Wirjanto TS (2004). The empirical role of the exchange rate on the crude-oil price formation. Energy Economics.

[CR84] Zhang YJ, Fan Y, Tsai HT, Wei YM (2008). Spillover effect of US dollar exchange rate on oil prices. Journal of Policy Modeling.

