# Correction to: The role of cryptocurrencies in predicting oil prices pre and during COVID-19 pandemic using machine learning

**DOI:** 10.1007/s10479-022-05078-4

**Published:** 2022-11-21

**Authors:** Bassam A. Ibrahim, Ahmed A. Elamer, Hussein A. Abdou

**Affiliations:** 1 Department of Management, Faculty of Commerce, Mansoura University, Mansoura, Egypt; 2 Brunel Business School, Brunel University London, Kingston Lane, UB8 3PH Uxbridge, London, UK; 3 Departmentof Accounting, Faculty of Commerce, Mansoura University, Mansoura, Egypt; 4 Faculty of Business & Justice, University of Central Lancashire, PR1 2HE Preston, UK

**Annals of Operations Research**

10.1007/s10479-022-05024-4

The original online version of this article was revised as typesetter overlooked author corrections during proofing.

Original article has been corrected.

